# An integrated method for optimized identification of effective natural inhibitors against SARS-CoV-2 3CLpro

**DOI:** 10.1038/s41598-021-02266-3

**Published:** 2021-11-23

**Authors:** Qi Liao, Ziyu Chen, Yanlin Tao, Beibei Zhang, Xiaojun Wu, Li Yang, Qingzhong Wang, Zhengtao Wang

**Affiliations:** Shanghai Key Laboratory of Compound Chinese Medicines, The MOE Key Laboratory for Standardization of Chinese Medicines, Institute of Chinese Materia Medica, Shanghai University of Traditional Chinese Medicine, Shanghai, China

**Keywords:** Computational biology and bioinformatics, Drug discovery

## Abstract

The current severe situation of coronavirus disease 2019 (COVID-19) caused by severe acute respiratory syndrome coronavirus 2 (SARS-CoV-2) has not been reversed and posed great threats to global health. Therefore, there is an urgent need to find out effective antiviral drugs. The 3-chymotrypsin-like protease (3CLpro) in SARS-CoV-2 serve as a promising anti-virus target due to its essential role in the regulation of virus reproduction. Here, we report an improved integrated approach to identify effective 3CLpro inhibitors from effective Chinese herbal formulas. With this approach, we identified the 5 natural products (NPs) including narcissoside, kaempferol-3-O-gentiobioside, rutin, vicenin-2 and isoschaftoside as potential anti-SARS-CoV-2 candidates. Subsequent molecular dynamics simulation additionally revealed that these molecules can be tightly bound to 3CLpro and confirmed effectiveness against COVID-19. Moreover, kaempferol-3-o-gentiobioside, vicenin-2 and isoschaftoside were first reported to have SARS-CoV-2 3CLpro inhibitory activity. In summary, this optimized integrated strategy for drug screening can be utilized in the discovery of antiviral drugs to achieve rapid acquisition of drugs with specific effects on antiviral targets.

## Introduction

Coronavirus disease 2019 (COVID-19) is an infectious disease caused by severe acute respiratory syndrome coronavirus 2 (SARS-CoV-2), which can lead to various symptoms including fever, cough, fatigue, shortness of breath, and loss of smell and taste^1^. Up to September 10, 2021, more than 223.2 million persons have been infected into COVID-19 across 192 countries or regions which resulted into 4,605,789 deaths^2^. Thus it will be urgent to seek for the effective treatment against COVID-19.

Since the outbreak of SARS-CoV-2, various antiviral compounds have been developed to treat COVID-19^3^. At present, mainstream antivirus research has been conducted on the mechanism of virus replication, and many effective compounds have been discovered. A series of antivirals being tested against SARS-CoV-2 such as PF-07304814^4^, remdesivir^5^, GC376^6^, apilimod^7^, nelfinavir^8^ and quinacrine^9^, displayed high antiviral activity in vitro. Some of them, such as remdesivir^10^, favipiravir^11^ and PF-07304814 are also under clinical investigation. However, no drugs have shown outstanding therapeutic effects in clinical trials. As the epidemic situation remains grim, there is still an urgent need for effective methods to discover valid antiviral drugs.

In addition to the mentioned chemical compounds, another important treatment approach is antiviral natural products (NPs) and herbal medicines. These herbal medicines as adjunctive treatment have been used to administrate the mild and moderate patients with coronavirus infection, including those caused by Middle East respiratory syndrome coronavirus (MERS-CoV), SARS-CoV and SARS-CoV-2^12–14^. Traditional Chinese medicine (TCM), as a material basis for the application of NPs and herbal medicines under the guidance of theory, have been found to be an effective treatment for COVID-19. Previously, the "three-medicines and three-prescriptions (TMTP)" strategy was recommended as a prescribed formula by the State Council of China because TMTP has exhibited the remarkable therapeutic effects and no side effects, especially against COVID-19^15^. TMTP mainly include *Jinhua Qinggan* granules, *Lianhua Qingwen* granules and capsules, *Xuebijing* injection, *Qingfei Paidu* decoction, *Huashi Baidu* decoction and *Xuanfei Baidu* decoction, which have presented good clinical efficacy in the treatment of COVID-19^16^. This strategy halts the progression of the disease and actively alleviates and improves symptoms during the early and middle stages^16,17^.

As a combination of several compound medicines, TMTP contains abundant molecules that make the antiviral mechanism unclear. It is necessary to apply an efficient method that quickly and effectively finds the basis of antiviral substances from this valuable natural molecular library. In recent years, the application of in silico technology in drug discovery has achieved prominent success^18,19^, supplying sophisticated tools for screening promising lead compounds, predicting potential protein catalytic sites or revealing the mode of protein–ligand interactions. During this pneumonia epidemic, studies have utilized the favored approaches that target SARS-CoV-2 with high-throughput screening of large-scale molecular databases and obtaining potential antiviral drugs^20,21^. With the advancement of computer technology, the combination of computer-aided drug design (CADD) and artificial intelligence (AI) research has become a valuable tool to accelerate the slow process of drug discovery and restraint the expansion of R&D costs, expand the applicable system and improve the level of automation, followed by the development of CADD-based multithreaded in silico screening technology^22–25^. Within the framework of above idea, we proposed a multimodule integrated approach aimed at improving the lead compound screening accuracy and greatly reducing the time cost by fully maximizing the advantages of each module to achieve a semiautomatic pipeline.

Based on the above purposes, an integration of CADD, machine learning (ML) and similarity-based clustering methodologies and experimental validation was adopted in our study as an accurate and efficient means to filter potential anticoronavirus candidates from TMTP database against 3CLpro, the key target of viral replication^26–29^. We expeditiously discovered 5 NPs inhibitors, which further supports that the integrated strategy can accurately and quickly achieve the purpose of obtaining promising lead compounds, suggesting its practicality and worthiness for further optimizing the application form and systems.

## Results

### Workflow construction and molecular data base constitutions

**Figure 1:**
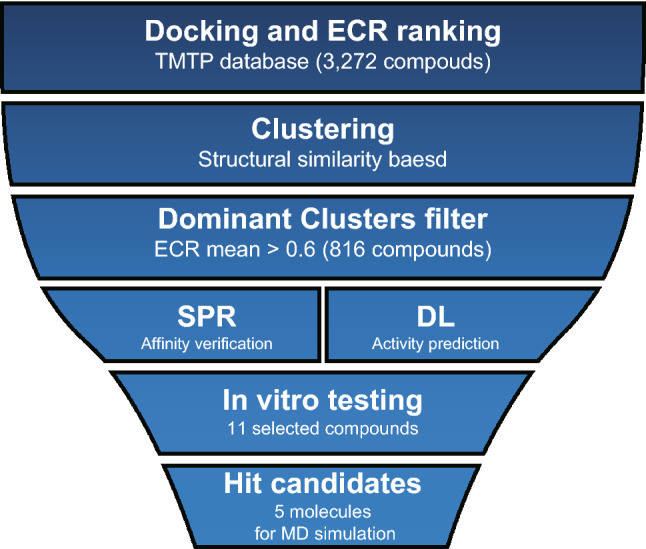
Flowchart represents the workflow of integrated structure-based anti-coronavirus NPs screening.

This integrated screening workflow is divided into four parts (Fig. 1). First, a molecular library was constructed, and the affinity ranking was obtained through molecular docking. Then, cluster analysis was performed to reveal the molecular characteristics of high-affinity clusters and extract the top clusters. Furthermore, combined with high-throughput methods applied to determine the binding affinity and predict the level of activity, the molecules with both excellent properties were finally verified experimentally to determine effective compounds.

In the foremost step, we retrieved 49 Chinese medicinal materials involved in the 6 compound herbal formula in TMTP (Supplementary Table 1). First, excluding gypsum, which is mainly inorganic salt, 5464 SDF files of each chemical ingredient related to 46 Chinese medicinal materials were obtained through TCMSP database, the molecules of Herba Rhodiolae^30^ and Rhizoma Areactylodis Lanceae^31^ were excavated from literature, then 8 and 13 compounds were added from PubChem respectively. Second, we removing duplicates from 5485 compounds. Finally, 3272 compounds were obtained, which is the TMTP molecular database. This library include the Chinese herbal compound prescriptions and the representative Chinese medicines from TMTP as well as the main chemical compositions. On the other hand, we collected 301 of SARS-CoV and 84 of SARS-CoV-2 3CLpro inhibitors, which was performed as comprehensively as possible. The former was used to build ML models, and the latter were treated as a test set. A complete list of the molecules and related information for 3272 TMTP compounds library, compound libraries and SARS-CoV and SARS-CoV-2 3CLpro inhibitors is given in the Supplementary Data 1.

### 20 clusters divided from the TMTP compound library by cluster analysis

To classify the structural similarity of high-affinity molecules to further narrow the range of lead compounds, a total of 8 combinations between similarities of fingerprint maps and different cluster agglomeration methods were individually used for cluster analysis. Based on the agglomerative coefficient from *agnes*, we found that the combination of Euclidean and Ward2 exhibited the highest value (agglomerative coefficient = 0.975) compared with that of the other groups, and the agglomerative coefficients of the 8 groups are listed in Supplementary Table 2. Thus, we adopted the Euclidean and Ward2 combination to plot a clustering that contained 20 clusters (k = 20) (Supplementary Fig. 1, Supplementary Table 3).

### Dominant clusters determined by means of molecular docking and ranking

Our molecular docking approach was used to obtain the binding ability of the TMTP molecular library (3272 molecules) with SARS-CoV-2 3CLpro, as well as the affinity score between positive inhibitors with SARS-CoV (301 of SARS-CoV 3CLpro inhibitors) or SARS-CoV-2 (301 of SARS-CoV and 84 of SARS-CoV-2 3CLpro inhibitors) 3CLpro for ML modeling. Docking analysis was carried out independently using the programs Autodock Vina, Glide, and MOE. Then, the exponential consensus ranking (ECR) strategy was implemented to reduce the number of false positives. This approach transformed docking scores of a single compound into a decimal number to indicate the comprehensive binding level for the target-ligand complex. Subsequent analyses were performed using ranking values instead of docking scores (Supplementary Data 1).

To compare the binding capacity to 3CLpro among clusters, we calculated and ranked the median, mean and quantile value, etc. of the molecular ranking in each cluster. Then, the dominant clusters were defined as those with a mean ranking value greater than 0.6 (Supplementary Table 4), and 9 dominant clusters were ultimately acquired. Among the 9 dominant clusters, the average ranking value of the royalblue cluster and brown cluster was greater than 0.7, indicating that these two clusters have higher target affinity.

### Combining binding affinity of SPR and inhibitory activity prediction by ML analysis to narrow the range of hit compounds

In current study, surface plasmon resonance (SPR) was used to rapidly identify molecules in the dominant clusters that have the ability to bind to SARS-CoV-2 3CLpro. As a result, 21 molecules demonstrated high affinity for 3CLpro (Supplementary Table 5). ML was applied in parallel with SPR analysis to predict the 3CLpro inhibitory efficiency of compounds in the dominant clusters and further eliminate the molecules that would be nonspecifically bound in the SPR analysis. As previously described, the collected information on 3CLpro inhibitors (301 SARS-CoV and 84 SARS-CoV-2 inhibitors), including IC_50_, pIC_50_, SMILES, and CID, is shown in Supplementary Data 2. After the molecular docking process, we acquired the docking scores and molecular ranking values between the SARS-CoV 3CLpro inhibitors and 3CLpro of SARS-CoV and SARS-CoV-2 using three different software programs. Overall, no significant difference between docking scores or molecular ranking values of SARS-CoV and SARS-CoV-2 was observed, which was ascribed to the high homology of the SARS-CoV and SARS-CoV-2 3CL proteins^32^. We calculated the similarity index (0.710) of two proteins binding or activity based on the docking matrix. Then, the predicted IC_50_ of SARS-CoV-2 3CLpro was computed based on the Eq. (2). By means of *Rcpi*, the molecular descriptors of 301 compounds were extracted as the quantitative structure (Supplementary Data 2). Thereafter, we constructed the quantitative relationship between structure and activity by random forest (RF) and support vector machine (SVM) training classification models. The activity of the 84 compounds for SARS-CoV-2 3CLpro was tested using the training model. To deal with the imbalance training datasets in the RF and SVM algorithm, we used the method of additional sampling that was conducted after resampling (usually to resolve class imbalances). The results of comparison among four resampling methods in these two algorithms had shown the area under curve (AUC) value of smote method of RF was higher than the other methods (RF: AUC_smote_ = 0.87, AUC_down_ = 0.83, AUC_regional_ = 0.85, AUC_weight_ = 0.86; SVM: AUC_smote_ = 0.81, AUC_down_ = 0.79, AUC_regional_ = 0.81, AUC_weight_ = 0.80). Thus, we incorporated the smote algorithm and cross-validation methods into the model in the train function. To further compare the RF and SVM analysis, the training model was tested with data of SARS-CoV-2 (81) and multiple evaluation metrics including the AUC, recall and precision value were calculated. We found that the AUC of receiver operating characteristic curve (ROC) in RF was higher than that of SVM (RF: AUC = 0.69, Precision = 0.24, Recall = 0.5; SVM: AUC = 0.59, Precision = 0.27, Recall = 0.30). Suggesting that the predicted inhibitory value calculated by the RF method was closer to the experimental value than that calculated by the SVM method.

Finally, we predicted the activity of 9 dominant clusters; here, a predicted value represented as possibility of positive result which have been computed with *predict* function and the selected type as *prob*, that greater than 0.5 was considered to have an inhibitory effect, and vice versa. A total of 156 compounds from 816 compounds in the dominant cluster were predicted to be active via ML analysis. A complete list of the predicted values can be found in Supplementary Data 3.

We combined the binding ability results from SPR analysis and the predicted activity results from ML analysis. The 11 NPs that have shown positive both SPR and ML analysis were considered potential inhibitor candidates and utilized for further experiments. Interestingly, these high-activity compounds were enriched in the brown, midnightblue and red clusters (Supplementary Table 6). In the above clusters, the brown cluster mainly contains flavonoids and their glycosides. The midnightblue cluster is composed of dammarane and oleanane or their derivative parent nucleus and corresponding glycosides. The compounds in the red cluster are composed of polyhydroxy conjugated systems such as hydroxytyrosol and caffeic acid to connected with sugar units. These types of compounds often exhibit a wide range of biological activities and have also been used in the field of anti-virus^33,34^.

### 5 NPs identifying as potent inhibitors of SARS-CoV-2 3CLpro in vitro

**Figure 2:**
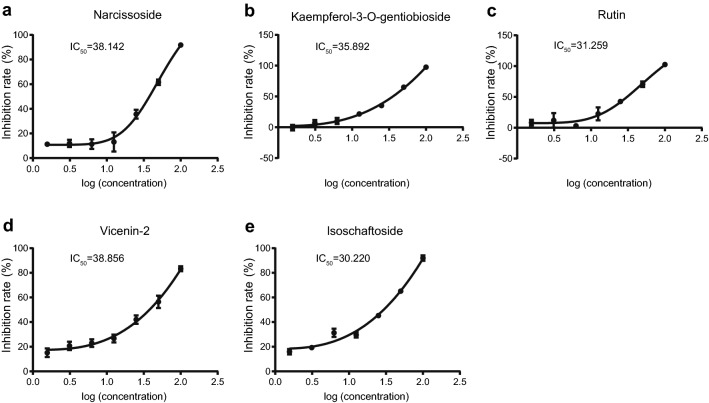
SARS-CoV-2 3CLpro in vitro dose–response inhibition assay and IC50 value detection. (**a**) Narcissoside. (**b**) Kaempferol-3-O-gentiobioside. (**c**) Rutin. (**d**) Vicenin-2. (**e)** Isoschaftoside.

**Figure 3:**
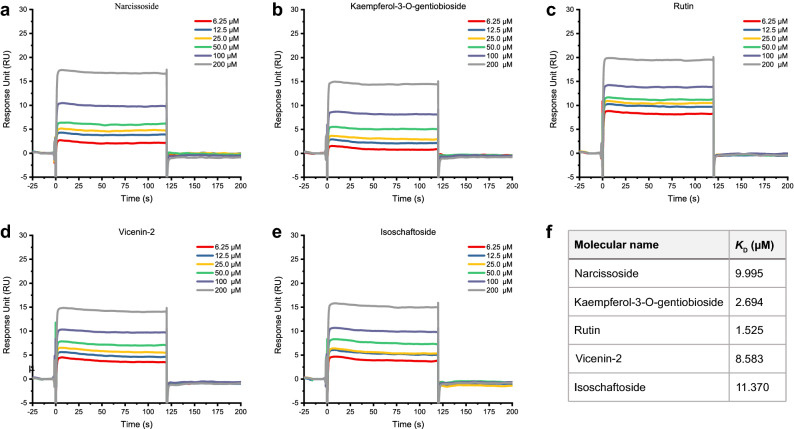
Kinetic binding curve of 5 3CLpro inhibitors measured by SPR experiment. **(a)** Narcissoside. (**b)** Kaempferol-3-O-gentiobioside. (**c)** Rutin. (**d)** Vicenin-2. (**e)** Isoschaftoside. (**f)** The dissociation equilibrium constant (*K*_D_) value of the five natural inhibitors.

Eleven compounds selected by the virtual screening and ML analysis were subsequently tested using the inhibition assay against SARS-Cov-2 3CLpro. After the initial screening, only five compounds at a concentration of 100 μM demonstrate over 50% inhibitory active against the enzyme. These compounds were able to achieve inhibition at lower concentrations. According to results shown in Fig. 2, narcissoside (MOL003686), kaempferol-3-O-gentiobioside (MOL012143), rutin (MOL000415), vicenin-2 (MOL001543) and isoschaftoside (MOL004958) presented IC_50_ values of 38.142, 35.892, 31.259, 38.856 and 30.220 μM, respectively. Remarkably, they are all flavonoids. The results of affinity screening by SPR showed that flavonoids accounted for 10 of the 21 compounds and their *K*_D_ values ranged from 1.525 to 12.46, exhibited strong affinity with 3CLpro (Fig. 3). As demonstrated in Table 1, the *K*_D_ values are well correlated with IC_50_ values. Notably, kaempferol-3-o-gentiobioside, vicenin-2 and isoschaftoside were reported as anticoronavirus candidates for the first time due to their inhibition of 3CLpro of SARS-CoV-2. They are very promising for further research to develop compounds with high inhibition efficiency.

**Table 1:** Summary of ranking, equilibrium dissociation constants (*K*_D_) and IC_50_ values for SARS-CoV-2 3CLpro inhibitors.

Compound	Molecular weight (Da)	Rank	*K*_D_ (µM)	IC_50_ (µM)
Narcissoside	624.544	0.744	9.995	38.142
Kaempferol-3-O-gentiobioside	610.518	0.861	2.694	35.892
Rutin	610.518	0.801	1.525	31.259
Vicenin-2	594.518	0.771	8.583	38.856
Isoschaftoside	564.492	0.765	11.370	30.220

### Molecular dynamics simulation revealed the stable binding mode of the 5 selected drugs with SARS-CoV-2 3CLpro

**Figure 4:**
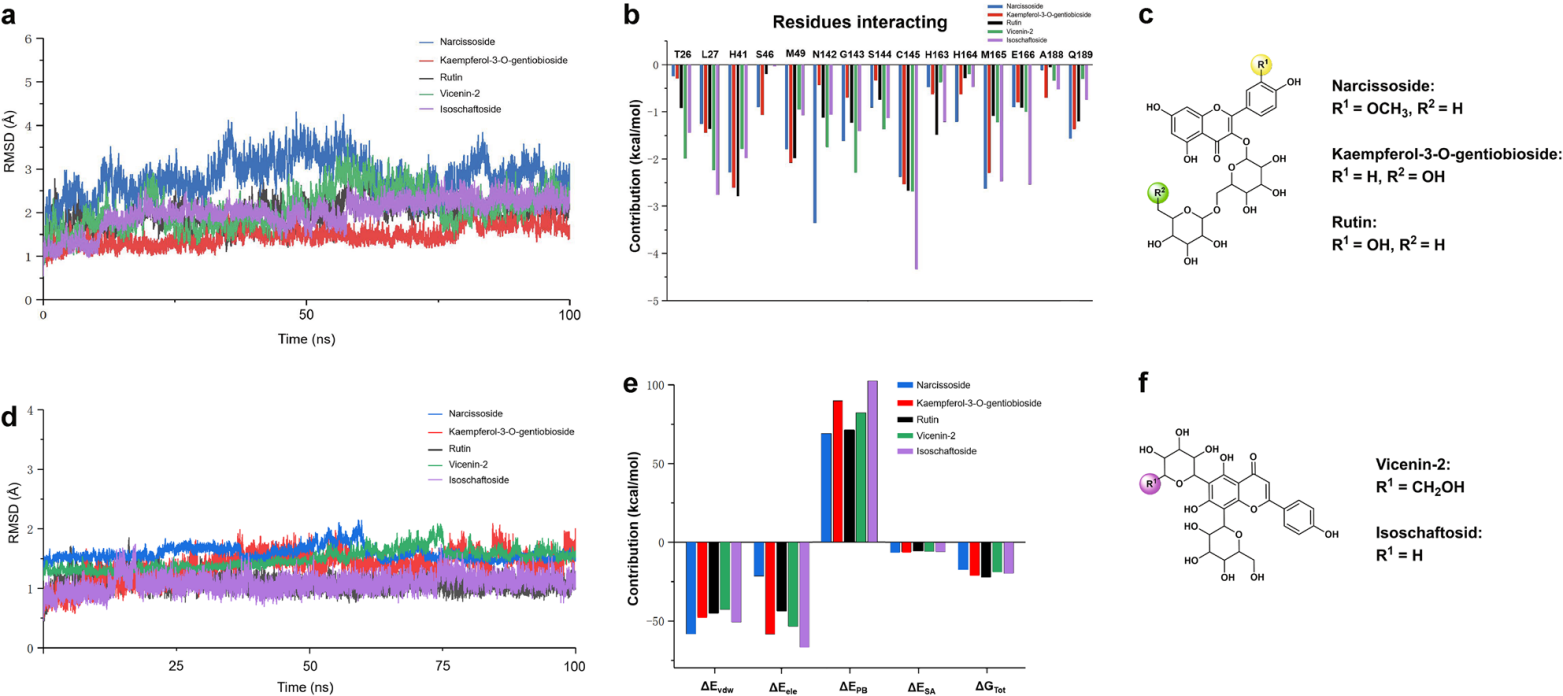
The RMSD values and contribution of various energy items to binding free energy in drug-3CLpro simulation. (**a**) Root mean square deviation (RMSD) of the 5 3CLpro-ligand complexes from the 100 ns MD simulations. Narcissoside (blue), kaempferol-3-O-gentiobioside (red), rutin (black), vicenin-2 (green) and isoschaftoside (Violet). (**b)** Residues with a high contribution to the total binding energy during the MD simulation of the 5 protein-inhibitor complexes. Narcissoside (blue), kaempferol-3-O-gentiobioside (red), rutin (black), vicenin-2 (green) and isoschaftoside (violet). (**c)** The general structure of narcissoside, kaempferol-3-O-gentiobioside and rutin. (**d)** Root mean square deviation (RMSD) of the 5 ligand from the 100 ns MD simulations. Narcissoside (blue), kaempferol-3-O-gentiobioside (red), rutin (black), vicenin-2 (green) and isoschaftoside (Violet). (**e)** The contribution of various energy items to the total binding energy of 3CLpro inhibitors. Narcissoside (blue), kaempferol-3-O-gentiobioside (red), rutin (black), vicenin-2 (green) and isoschaftoside (violet). **f** The general structure of vicenin-2 and isoschaftoside.

The dynamic binding interactions of the five compounds with inhibitory activity were analyzed, and 100 ns molecular dynamics (MD) simulations of ligand–protein complexes were performed. The root mean square deviation (RMSD) of the ligand trajectory was analyzed, revealing that the complexes rapidly reached equilibrium within the first 5 ns of the simulation (Fig. 4a), with each value lying between 1.5 and 3.5 Å. Narcissoside and vicenin-2 fluctuated greatly, indicating a flexible bingding to the active site of 3CLpro. In contrast, compounds kaempferol-3-O-gentiobioside, rutin, and isoschaftoside are more fixed. On the other hand, the degree of configuration change of these compounds in the binding pocket is relatively stable, and the RMSD is less than 0.5 (Fig. 4d).

To explore the binding affinity of each ligand to 3CLpro, the binding free energy was calculated based on MM/PBSA (Table 2). Van der Waals (ΔE_vdW_) and electrostatic (ΔE_ele_) interactions make major contributions to the binding free energy (Fig. 4e). We observed that rutin exhibited the highest binding affinity to 3CLpro, followed by kaempferol-3-O-gentiobioside, isoschaftoside, vicenin-2 and narcissoside**.** Analysis of the energy decomposition results of the five compounds suggested that the residues Thr25, Thr26, Ley27, His41, Cys44, Tgr45, Ser46, Met49, Asn142, Gly143, Cys145, His163, His164, Met165, Asp187 and Gln189 mainly contributed to hydrophobic and electrostatic interactions in the 3CLpro-ligand complex (Supplementary Table 7).

**Table 2:** The results of molecular MM/PBSA free energy calculation (kcal/mol) and relevant ranking.

Compound	ΔE_vdw_	ΔE_ele_	ΔE_PB_	ΔE_SA_	ΔG_Tot_	Rank
Narcissoside	− 58.32	− 21.59	69.90	− 6.30	− 16.31	0.744
Kaempferol-3-O-gentiobioside	− 47.22	− 58.54	90.81	− 5.89	− 20.84	0.861
Rutin	− 45.20	− 43.78	72.26	− 5.59	− 22.31	0.801
Vicenin-2	− 42.68	− 53.40	83.16	− 5.80	− 18.72	0.771
Isoschaftoside	− 50.61	− 66.58	103.47	− 5.95	− 19.67	0.765

**Figure 5:**
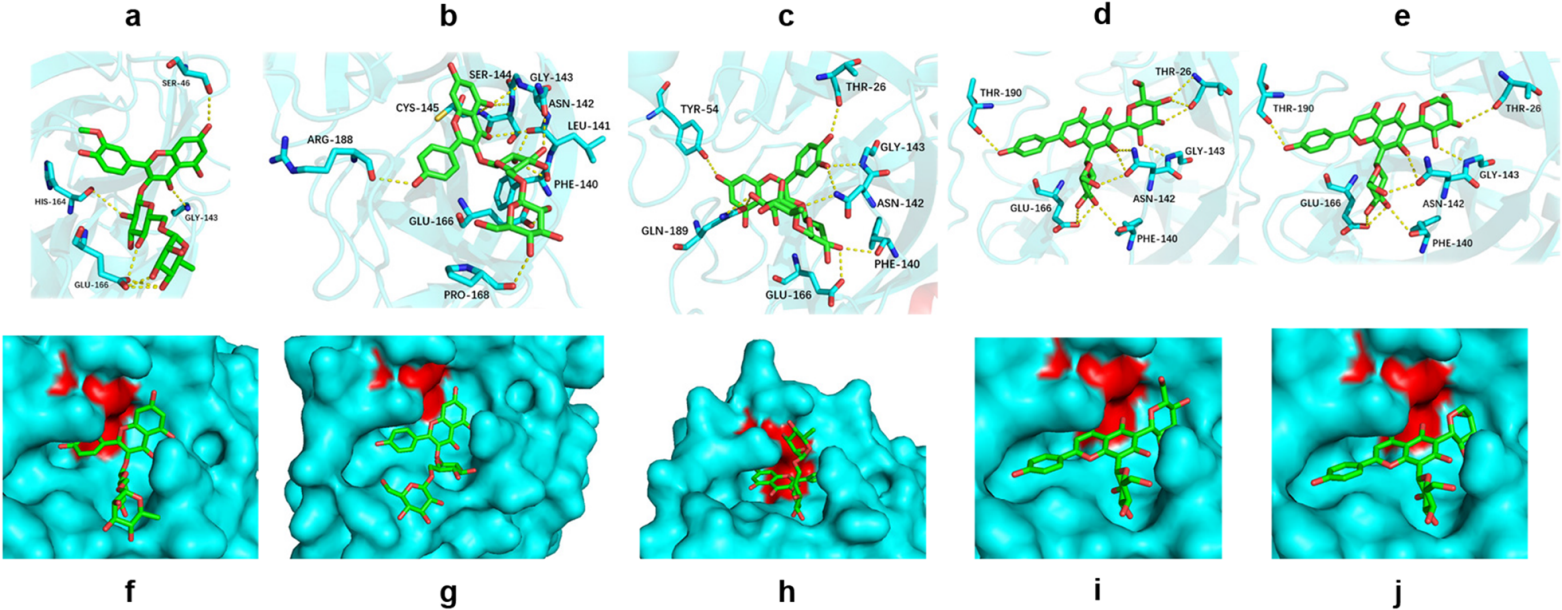
Binding mode of 5 NPs inhibitors to SARS-CoV-2 3CLpro. The protein and ligand are shown in gray and green, respectively. H-bond interactions are demonstrated by yellow dotted lines and residues forming H-bonds are shown in cyan. The surface of the protein is represented in cyan. The residues His41 and Met49 are highlighted in red to show the binding posture of the inhibitor. (**a)** Binding interaction of narcissoside. (**b)** Binding interaction of kaempferol-3-O-gentiobioside. (**c)** Binding interaction of rutin. (**d)** Binding interaction of vicenin-2. (**e)** Binding interaction of isoschaftoside. (**f)** Binding posture of narcissoside. (**g)** Binding posture of kaempferol-3-O-gentiobioside. (**h)** Binding posture of rutin. (**i)** Binding posture of vicenin-2. (**j)** Binding posture of isoschaftoside.

Specifically, from the analysis of the binding interactions, narcissoside showed the least electrostatic interaction (− 21.59 kcal/mol), forming hydrogen bonds with Ser46, Gly143, His164, and Glu166. Kaempferol-3-O-gentiobioside forms multiple hydrogen bonds with Phe140, Leu141, Asn142, Gly143, Ser144, Cys145, Glu166, Pro168, and Arg188. Rutin forms hydrogen bond interactions with Thr26, Tyr54, Phe140, Asn142, Gly143, Glu166, and Gln189. Vicenin-2 demonstrated the highest number of H-bonds, forming hydrogen bonds with Thr26, Phe140, Asn142, Gly143 and Glu166. In the analysis of binding energy with isoschaftoside, the contribution of electrostatic interactions to the total binding energy was − 66.58 kJ/mol, which was highest among the 5 compounds, forming H-bond interactions with Thr26, Tyr54, Phe140, Asn142, Gly143 and Glu166, Gln189. The above analysis suggested that flavonoid glycosides provided higher flexibility after forming chains with sugars because of their rotatable bonds, which can bind into pockets and form abundant hydrogen bonds with some key residues. From the perspective of amino acid energy decomposition (Fig. 4b), the compound has a strong interaction with His41, Met49, and Cys145. His41 and Met49 are also the active site residues of 3CLpro^35^. To facilitate the analysis, we first colored the region of the residues His41 and Met49, and then divided the five flavonoids into two categories according to their structural similarity. The active cavity of 3CLpro presented strong hydrophobicity, while the aromatic ring of the flavonoid aglycone provided the main hydrophobic energy contribution in the site. For type A (Fig. 4c), narcissoside, as the only inhibitor with methoxy group. The group has the function of enhancing hydrophobic action of ligand (-58.32 kcal/mol), making the benzene ring easily inserting into the hydrophobic region of the cavity (Fig. 5a,f), resulting in the overall structure extending outside of the cavity and reducing the interaction with residues, eventually reducing the contribution of the binding free energy. In contrast, the flavonoid skeleton of kaempferol-3-O-gentiobioside is close to the cavity (Fig. 5b,g). Furthermore, rutin is inserted into the cavity (Fig. 5c,h), which makes the binding tighter and presents the lowest binding free energy (Table 2). For type B (Fig. 4f), the overall structure shifted in the active pocket due to prolongation of the rigid flavonoid part in vicenin-2 (Fig. 5d,i) and isoschaftoside (Fig. 5e,j), resulting in the distance from the active site being farther than that for kaempferol-3-O-gentiobioside and rutin. However, they did not demonstrate much difference in their total binding free energies.

Notably, from the analysis of the binding interaction, with the key residues, we observed that the interaction strength between His41 and Met49 with the ligand was positively correlated with the affinity of the ligand and 3CLpro binding. In addition, the total energy (Supplementary Table 8) of residues based on the region of His41 to Met49 also exhibited this rule. On the other hand, ΔGTot calculated by MM/PBSA also matched the rank of the molecule (Table 2). The clear binding pattern and significant inhibitory activity of these five flavonoids against 3CLpro indicated that they are promising candidates for anti-SARS-CoV-2 activity. These results prove the correctness of our screening strategy.

## Discussion

To fight the epidemic and obtain effective antiviral drugs from a reservoir of herbal medicines, we designed an integrated pipeline workflow for NP screening. Our integrated strategy combining each submodule into the best workflow and fulfilling the optimization of function therefore exhibited a positive impact from rapid and accurate acquisition of lead compounds to subsequent structural optimization guidance. It is worth noting that the selection and optimization of submodules can be flexibly changed; this integrated strategy may not be limited to NPs screening. Furthermore, this strategy has the potential to derive automated pipelines from prototype workflows to improve the convenience of use while ensuring the accuracy of screening.

The screening pipeline was based on the binding affinity between molecules and targets. To avoid the affinity error caused by limitations of an algorithm of a single software which eliminate compounds that have true binding ability, we selected 3 commonly used docking software programs and converted docking scores into rankings of affinity trends by means of consensus analysis. Attributed to an "or" condition, the ECR assay can reduce the impact of extreme values in software scoring^36^, which achieved the fault tolerance of the discrepancy from different scoring functions, that it is very suitable for the research strategy of our study.

When confronted with a large compound library, effective cluster analysis can directly reflect the structural characteristics of molecules or clusters with high affinity and indicate the types of functional groups with high contribution to the corresponding complexes. This helps to guide the subsequent structural optimization and quickly eliminate the set of compounds with poor binding ability to the target. Consequently, similarity-based compound clustering is crucial in pipelines. Among the hierarchical and nonhierarchical clustering methods, Ward's and Jarvis-Patrick are known to be effective algorithms for chemical structure clustering^37,38^. As expected, in the course of practice, Jarvis-Patrick produced too many singletons and a small number of large clusters^39^, which is completely inconsistent with our requirements. For the similarity calculation, Tanimoto coefficients and Euclidean distance are the most widely used to evaluate how similar two molecules are to each other^40,41^. Then, we combined them with 4 commonly used hierarchical clustering algorithms and compare the calculated corresponding 8 sets of agglomerative coefficient. Thereafter, we found that Euclidean and Ward2 is the best matches to obtain a cluster of uniform internal structure characteristics, and the number of clusters can also be optimized for specific systems. In summary, this high-precision clustering is suited for but not limited to natural products, and it is worthy of promotion.

As an effective tool for predicting the structure–activity relationship, ML has been widely used in threaded approach^42,43^. The present ML-based activity prediction was capable of describing active molecules from the TMTP database even with a small-volume training set (301 compounds). We calculated the predicted value of SARS-CoV-2 3CLpro from the collected SARS-CoV 3CLpro inhibitory activity according to the similarity matrix between docking data of two target proteins. To build an effective model, we increased molecular character number of the training compound as an independent variable and covered the docking data. Moreover, we compared the predictive accuracy of the SVM and RF methods integrated into the cross validation analysis and found that the AUC value of random forest was higher than SVM, which provided the evidence for the candidates drug discovery by RF-based activity prediction.

Current cluster analysis accurately placed flavonoids into a subset and further obtained the 5 compounds with inhibitory activity of 3CLpro. As compounds characterized by 2-phenyl-benzyl-γ-pyrone nucleus, flavonoids are particularly valuable NPs that possess anti-inflammatory^44^, antioxidant^45^, anti-microbial^46^, and even antiviral activities. In a study of inhibitors against coronavirus, rhoifolin, pectolinarin, herbacetin^47^ and amentoflavone^48^ were demonstrated to block the function of SARS-CoV 3CLpro. The glycoside derivatives of kaempferol also proved to be virus release inhibit agents by blocking the 3a channel^49^. During the COVID-19 outbreak, narcissoside^50^ and rutin^29^ also exhibited the inhibitory effects on main protease of SARS-CoV-2, which is confirmed the practicality of our screening strategy.

The limitations of this study have been presented. First, on account of the few reports of tested compounds, a relatively small sample training set in the ML analysis was available. When more positive drugs are involved, the accuracy of ML activity prediction can be further improved. Second, the 5 NPs have not been evaluated the antiviral activity against SARS-CoV-2 by in vivo experimental and preclinical data due to the powerful invasion ability of SARS-CoV-2.

In conclusion, this study successfully employed an integrated screening strategy to identify 5 potential inhibitors of SARS-CoV-2 3CLpro from a NPs library composed of clinically effective herbal medicines. On the basis of this research, further research is worth pursuing to produce derivatives that can produce better inhibitors. The high efficiency and accurate characteristics of this strategy greatly shorten the hit cycle of lead compounds in the process of drug discovery for acute diseases and accelerate the process of drug development. We recommend that this integrated screening strategy be applied to other targets that urgently need effective drugs.

## Methods

### Construction of TMTP chemical constituent databases

Except for gypsum whose main component is inorganic salt, the SDF files of each chemical ingredient related to 46 Chinese medicinal materials were obtained through TCMSP database (Traditional Chinese Medicine Systems Pharmacology Database and Analysis Platform, http://lsp.nwu.edu.cn/tcmsp.php). By consulting the related literature of Herba Rhodiolae and Rhizoma Areactylodis Lanceae, SDF files were downloaded through PubChem respectively. The TMTP molecular database was obtained after the database deduplication. Then, the *OpenBabel* toolkit was used to convert the mol2 file of each molecule into unified SDF, pdbqt and SMILES file formats to prepare for molecular docking. Subsequently, we retrieved the absorption, distribution, metabolism and excretion (ADME) properties data from TCMSP database, containing molecular weight (MW), oral bioavailability (OB), number of hydrogen-bond acceptors (HBA), number of hydrogen-bond donors (HBD), etc. Other molecular descriptors, including atom additive logP (ALogP), atom molar refractivity values (AMR), and topological polar surface area (TPSA), were calculated based on the *Rcpi* in the R platform. All the discriptors were listed in Excle table. After deduplicating the SMILES file, the retained molecular discriptors entries and SDF files constituted a compound library for subsequent analysis.

### Molecular docking

To accurately predict docking poses, three different molecular docking programs, AutoDock Vina^51^ (version 1.1.2), Maestro (version 11.4, Schrödinger, LLC, New York, NY, 2021), and molecular operating environment software (MOE, Chemical Computing Group, version 2019.0101), were used to detect the binding capability between diverse compounds and SARS-CoV or SARS-CoV-2 3CLpro. The protein and ligand were prepared for the docking process. First, the crystal structural file of SARS-CoV and SARS-CoV-2 3CLpro were downloaded from protein database (PDB ID 3V3M^52^, 6LU7^53^). For the target protein, the preparation included carrying out the assign bond orders, hydrogenation, treatment of disulfide bonds, metal ions, and removal of water molecules, heteroatoms with default settings in three software. For ligand preparation, apart from AutoDock Vina which minimized the compound’s energy in Chem3D software, Maestro and MOE were carried out with inner LigPrep and Energy Minimize protocol respectively, to generated the correct form, and all the hydrogen atoms and the torsion information were added. To ensure the uniformity of different software at the docking position, the binding site box of 3CLpro coincided with binding site of the original inhibitors. Docking analysis was conducted with default protocol in AutoDock Vina, the Extra precision (XP) was used in GlideScore scoring functions^54^, and the Induced Fit module was chosen in MOE. The PyMOL Molecular Graphics System (version 2.0, Schrödinger, LLC) was used to visualize the docking posture of compounds at the binding pocket of 3CLpro.

### Consensus analysis

To combine results from several docking programs, we adopted ECR strategy proposed by Karen Palacio-Rodríguez et al.^36^ (2019) to assigned a rank to each molecule based on the docking scores of the molecules provided by different docking program. As shown in Eq. (1), ${\upsigma }$ represented the expected value of an exponential distribution and was assigned to be 10. $s_{n}$ referd to the compound docking score given by each software. The ultimate rank value of each molecule was defined as the sum of all the exponential scores $p\left( {s_{n} } \right)$, which was a positive correlation between the rank value and affinity of compound against target protein.

1 $$Rank = \mathop \sum \limits_{n} p\left( {s_{n} } \right) = \frac{1}{\sigma }\mathop \sum \limits_{n} {\text{exp}}\left( { - \frac{{s_{n} }}{\sigma }} \right)$$

### Cluster analysis

To identify homogeneous and distinct groups, or similar objects in TMTP NPs datasets, we performed clustering analysis with the *ChemmineR* and *WGCNA* packages. First, we collected the SDF file of each molecule produced by the OpenBabel software. By means of *readMolFromSDF*, we converted the SDF files into mol files. The SDF files were loaded into the *ChemmineR* for calculating atom pair fingerprints (APfp) of all compounds, which were used for calculate the structural similarity between the different compounds^55,56^. We adopted the Tanimoto coefficients and Euclidean distance for computing distance or dissimilarity metrics based on the fingerprint of TMTP natural products. In this proccess, we mainly utilized the sdf2ap and fpSim function from *ChemmineR* package. Firstly, we extracted atom pair fingerprints from 3272 NPs sdf files through *sdf2ap* function. Then, we calculated pairwise compound structure comparisons from fingerprint dataset using *fpSim* function. The fingerprint-based Tanimoto or Euclidean similarity matrix were computed and the other parameters were set as default values. To classify the different compounds into a series of the relative number of clusters, we concentrated on single, complete, average, and Ward's algorithms to map the strength of the clustering results. Additionally, the dendrograms of 8 *hclust* approaches were plotted, and the *agnes* from *dendextend* computing the agglomerative coefficient was used to measure the amount of clustering structure found (values closer to 1 suggest a strong clustering structure). To clearly delineate the different clusters, we integrated the *WGCNA*, which can effectively assign different modules and are represented as visualized colors. The functions of *cutreeDynamic* and *labels2colors* were further used to investigate the best clustering results.

### Surface plasmon resonance analysis

The binding studies were performed at 25 °C on a Biacore T200 instrument at a flow rate of 30 μL/min in running buffer composed of PBS (pH 7.4) and 3 mM EDTA. CM7 chips activated in a 10 min injection procedure with a mixture of EDC (1-ethyl-3-(3-dimethylaminopropyl)-carbodiimide)/NHS (Nhydroxysuccinimide) (0.2 M/0.05 M) and immobilized with anti-histone antibody until levels of immobilization were between 15,000 and 20,000 RU. The chip was then deactivated with a 7 min injection of 1 M ethanolamine (pH 8.0). The 3CLpro-his protein was then applied on the chip to reach typically levels between 4000 and 5000 RU. The binding activity and stability of proteins to ligands were tested at the end of each compound injection. The association and dissociation phases of tested ligands were monitored for 120 s each. The final binding experiment with small molecule ligands was performed in the above running buffer supplemented with 5% DMSO. PBS, EDTA, CM7 chip, EDC, NHS and ethanolamine were obtained from GE, 3CLpro-his was obtained from Kangma- Healthcodea.

### Machine learning analysis

3CLpro Positive inhibitors of SARS-CoV and SARS-CoV-2 for individual modeling and validation via ML were acquired from the literature, related IC_50_ were collected, and the SDF files was downloaded from Pubchem or generated by ChemDraw, as well as SMILES files. Then, each 3CLpro inhibitor of SARS-CoV was docked with the 3CLpro of SARS-CoV and SARS-CoV-2 separately, as described in the docking procedure above, rank of a molecule was constructed from the docking score matrix. To eliminate the inconvenience of calculation and data comparison caused by the orders of magnitude difference between the data, pIC_50_ was applied in calculation instead of IC_50_. After that, the inhibitory efficiency in the data set was converted to two classes: active (pIC_50_ ≥ 6) and inactive (pIC_50_ < 6). Then, the similarity index (SI) between docking score of compound and 3CLpro complex in SARS-CoV and SARS-CoV-2 was calculated via *SMI* from *MatrixCorrelation*^57^. To build the predicted model, we proposed the Eq. (2) to transform the SARS-CoV IC_50_ to predicted IC_50_ of the SARS-CoV-2 inhibition efficiency. Subsequently, a classification model based on the quantitative structure and activity relationship of SARS-CoV-2 was constructed.

2 $$pIC_{{50^{pre = } }} SI \times pIC_{{50^{SARS - CoV} }}$$

The inhibitory compounds targeting SARS-CoV-2 3CLpro and the related IC_50_ were validated as a test data set. By means of *Rcpi*, we extracted the molecular descriptors of the validated compounds, including ALogP, square of AlogP (ALogp2), AMR, atomic polarizabilities (apol), eccentric connectivity index (ECCEN), topological polar surface area (TopoPSA), MW, weiner path number (WPATH), weiner polarity number (WPOL) and the sum of the squares of atom degree over all heavy atoms (ZagrebIndex). The molecular descriptors and docking value of the training data sets were input as independent variables, and the predicted IC_50_ values were input as dependent variables. We selected the random forest (RF) and support vector machine (SVM), which are widely used ML methods^58,59^. We established the machine learning analysis by the *caret* package. Repeated cross validation (times = 5, fold = 10) were used as the cross-validation strategies. And we optimized the important parameters including sigma, C and weight which were used to select the optimal model. The main hyperparameters of SVM contain sigma (sigma = 0.1), cost (C = 1) and weight (weight = 3) while the hyperparameters of RF include mtry (mtry = 2). Meanwhile, considering that the imbalanced training dataset, we explored different algorithms, including original, weighted, down and smote algorithms, in the training stage. The accuracy of two classification model was evaluated by the AUC value of ROC. The recall and precision values were calculated with *confusionMatrix* function. Based on the AUC value, we selected the optimal methods to predict the inhibition efficiency of natural products from dominant clusters.

### SARS-CoV-2 3CLpro inhibition assay

The inhibition assay of SARS-CoV-2 3CLpro was carried out based on the reported method^60^. In the initial step, 0.5 µg of SARS-CoV-2 3CLpro was preincubated with 100 µL of 200 µM test compound at room temperature for 15 min. Then, the reaction was triggered after the addition of 10 µM Dabcyl-KTSAVLQSGFRKME-Edans (GL Biochem). The fluctuation of fluorescence intensity was monitored on a GENios microplate reader (Tecan), where the excitation wavelength was 340 nm and the emission wavelength was set to 490 nm. Control reactions were performed under the same condition, but the compounds or enzymes were excluded from the reaction system. The median inhibitory concentration (IC_50_) values against SARS-CoV-2 3CLpro was calculated by nonlinear regression analysis via GraphPad Prism 7.03 (GraphPad Software, San Diego, CA, USA).

### Molecular dynamics simulation and binding free energy calculation

MD simulations of the screened natural inhibitors of 3CLpro were performed with Amber14^61^ to evaluate their binding interaction patterns with 3CLpro. The protein–ligand complexes were used as the initial structure for subsequent MD simulations. The ligand and protein were treated with General Amber Force Field (GAFF^62^) and FF14SB^63^ respectively. For the amino acid residues of the protein, the default protonation state in Amber14 was adopted, and the hydroprocessing was carried out using *tleap* module. By means of the Gaussian09 software package^64^, the Lee–Yang–Parr correlation functional (B3LYP)/6-31G** was carried out to optimized all inhibitors. The restrained electrostatic potential (RESP) charges as partial charges for molecules were calculated by fitting with the standard RESP procedure implemented in the *Antechamber* module of the Amber14. And molecular dynamics simulations for all complexes employ the PMEMD program in Amber14. After adding counter ions to each complex to maintain the neutrality of the system, the entire system was contained to a TIP3P rectangular water box. Furthermore, energy minimization was performed by steepest descent method of 2500 steps and conjugate gradient method of 2500 steps. Subsequently, same methods were used to optimize the unconstrained system. The Particle mesh Ewald (PME) was performed in the MD simulation to deal with the long-range electrostatic interaction, and the SHAKE algorithm was used to constrain all the bonds connected to the hydrogen atom and the time step was set to 2 fs. Then set a cutoff value of 10 Å for non-bonding interactions. The constrained whole system was heated from 0 to 300 K in 60 ps at a constant volume, subsequently, the solvent density was balanced in a constant pressure and thermostatic system (T = 300 K, P = 1 ATM). Followed by 100 ns of MD simulations control at constant pressure and frames were saved at 1 ps intervals (50,000 frames totally) for subsequent MM/PBSA analysis at last. In order to obtain the RMSD, the trajectory was generated from MD simulation via *cpptraj* module then analyzed with the VMD (1.9.3) program^65^. OriginPro (Version 2021 Learning Edition. OriginLab Corporation, Northampton, MA, USA.) was used for plot.

## Supplementary Information

Supplementary Information 1.

Supplementary Information 2.

Supplementary Information 3.

Supplementary Information 4.

Supplementary Information 5.

## Data Availability

The authors declare that all data supporting the findings of this study are available within the paper and its supplementary information files.

## Code availability

The authors declare that all code supporting the findings of this study are available from the corresponding author on reasonable request.
