# Supplementary material for: An integrated method for optimized identification of effective natural inhibitors against SARS-CoV-2 3CLpro
Source: Sci Rep. 2021 Nov 23;11:22796. doi: 10.1038/s41598-021-02266-3 (PMC8611036; doi:10.1038/s41598-021-02266-3)
Supplement: Supplementary file 1 — Supplementary Information 1. [file 41598_2021_2266_MOESM1_ESM.pdf]

Description of Additional Supplementary Files

File Name: Supplementary Data 1

Description: The compound information was summarized in the tables including TMTP molecular library, DL training set (SARS-CoV 3CLpro inhibitors) and test set (SARS-CoV-2 3CLpro inhibitors). In addition, the docking scores of TMTP library compounds with SARS-CoV-2 3CLpro, training set compounds with SARS-CoV and SARS-CoV-2 3CLpro and test set compounds with SARS-CoV-2 3CLpro, the corresponding ECR ranking scores were also included.

File Name: Supplementary Data 2

Description: In the excel tables, training set and test set molecular descriptors were calculated for DL modeling and optimization.

File Name: Supplementary Data 3

Description: The compound activity data predicted by DL. Compounds of test sets and dominant clusters were analyzed respectively through DL to predict their inhibitory activity against SARS-CoV-2 3CLpro. The value of "Pred. act." is proportional to the intensity of the potential inhibitory activity, and it is considered as no inhibitory activity if it is less than 0.5.
