# Supplementary material for: An integrated method for optimized identification of effective natural inhibitors against SARS-CoV-2 3CLpro
Source: Sci Rep. 2021 Nov 23;11:22796. doi: 10.1038/s41598-021-02266-3 (PMC8611036; doi:10.1038/s41598-021-02266-3)
Supplement: Supplementary file 5 — Supplementary Information 5. [file 41598_2021_2266_MOESM5_ESM.pdf]

# Supplementary Information

## An integrated method for optimized identification of effective natural inhibitors against SARS-CoV-2 3CLpro

Qi Liao[1 *], Ziyu Chen[1 *], Yanlin Tao[1], Beibei Zhang[1], Xiaojun Wu[1], Li Yang[1 #], Qingzhong Wang[1 #], Zhengtao Wang

[1]Shanghai Key Laboratory of Compound Chinese Medicines, The MOE Key Laboratory for Standardization of Chinese Medicines, Institute of Chinese Materia Medica, Shanghai University of Traditional Chinese Medicine, Shanghai, China

* These authors contributed to the work equally

[#] Corresponding Author

Li Yang: yl7@shutcm.edu.cn
Qingzhong Wang: wangqingzhong3@gmail.com

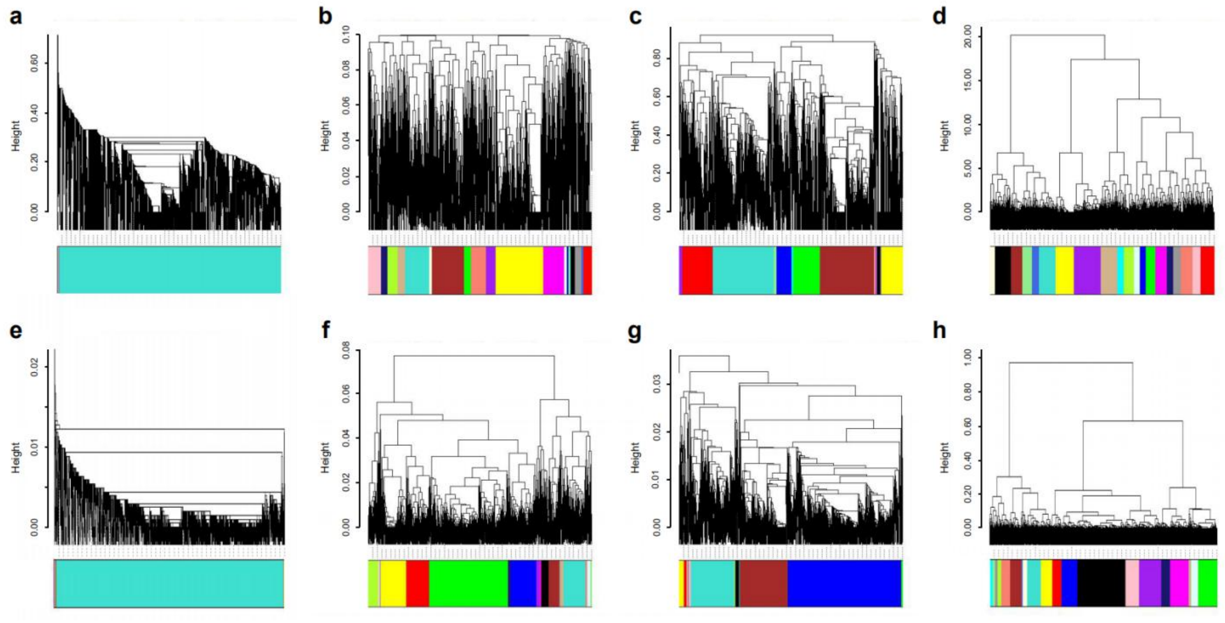


**Supplementary Figure 1.** The dendrogram of the 8 combination between similarities of fingerprint maps and different cluster agglomeration. **a** Group of tanimoto and single. **b** Group of tanimoto and complete. **c** Group of tanimoto and average. **d** Group of tanimoto and ward2. **e** Group of euclidean and single. **f** Group of euclidean and complete. **g** Group of euclidean and average. **h** Group of euclidean and ward2.

| **Chinese medicine prescription** | **Number of constituent** | **Latin name of corresponding herb** |
|---|---|---|
| Jinhua Qinggan granules | 12 | Flos Lonicerae, Gypsum Fibrosum, Gypsum Fibrosum, Semen Armeniacae Amarae, Radix Scutellariae, Fructus Forsythiae, Bulbus Fritillariae Thunbergii, Rhizoma Anemarrhenae, Fructus Arctii, Herba Artemisiae Annuae, Herba Menthae, Radix Glycyrrhizae |
| Lianhua Qingwen granules and capsules | 13 | Radix Isatidis, Fructus Forsythiae, Flos Lonicerae, Rhizoma Dryopteris Crassirhizomatis, Herba Ephedrae, Semen Armeniacae Amarae, Herba Houttuyniae, Herba Pogostemonis, Herba Rhodiolae, Radix et Rhizoma Rhei, Radix Glycyrrhizae, Gypsum Fibrosum, Herba Menthae |
| Xuebijing injection | 5 | Flos Carthami, Radix Paeoniae Rubra, Rhizoma Chuanxiong, Radix Salviae Miltiorrhizae, Radix Angelicae Sinensis |
| Qingfei Paidu decoction | 21 | Herba Ephedrae, Semen Armeniacae Amarae, Ramulus Cinnamomi, Rhizoma Alismatis, Grifola, Rhizoma Atractylodis Macrocephalae, Poria, Radix Bupleuri, Radix Scutellariae, Rhizoma Pinelliae, Rhizoma Zingiberis Recens, Radix Asteris, Flos Farfarae, Rhizoma Belamcandae, Herba Asari, |

| | | |
|---|---|---|
| | | Rhizoma Dioscoreae, Fructus Aurantii Immaturus, Tangerine Peel, Herba Pogostemonis, Radix Glycyrrhizae, Gypsum Fibrosum |
| Huashi Baidu decoction | 14 | Herba Ephedrae, Semen Armeniacae Amarae, Radix Paeoniae Rubra, Semen Lepidii, Rhizoma Pinelliae, Poria, Fructus Tsaoko, Herba Pogostemonis, Rhizoma Atractylodis, Radix Astragali, Cortex Magnoliae Officinalis, Radix et Rhizoma Rhei, Gypsum Fibrosum, Radix Glycyrrhizae |
| Xuanfei Baidu decoction | 13 | Herba Ephedrae, Semen Armeniacae Amarae, Semen Coicis, Rhizoma Areactylodis Lanceae, Herba Pogostemonis, Herba Artemisiae Annuae, Rhizoma Polygoni Cuspidati, European Verbena, Rhizoma Imperatae, Semen Lepidii, Exocarpium Citri Grandis, Radix Glycyrrhizae, Gypsum Fibrosum |

**Supplementary Table 1. The summary of prescription and herbal medicines in TMTP.**

| Entry | Metrics method | Clustering method | Agglomerative coefficient |
|---|---|---|---|
| 1 | Tanimoto | Single | 0.129 |
| 2 | Tanimoto | Complete | 0.196 |
| 3 | Tanimoto | Average | 0.205 |
| 4 | Tanimoto | Ward2 | 0.943 |
| 5 | Euclidean | Single | 0.656 |
| 6 | Euclidean | Complete | 0.760 |
| 7 | Euclidean | Average | 0.674 |
| 8 | Euclidean | Ward2 | 0.975 |

**Supplementary Table 2. Agglomerative coefficient of 8 combined clustering methods.**

*Agnes* function from *dendextend* was computed for the agglomerative coefficient to indicate the degree of superiority of the clustering method (values closer to 1 suggest a strong clustering structure).

| Color | n | Molecular type | Representation molecule | Chemical structure | Molecular name |
|---|---|---|---|---|---|
| royalblue | 19 | Polygallic acid linking central sugar unit | MOL010175 | 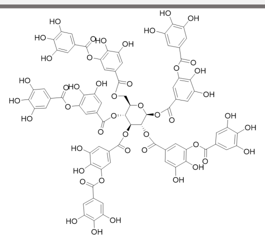 | Tannic acid |
| brown | 165 | Flavonoid glycosides | MOL003686 | 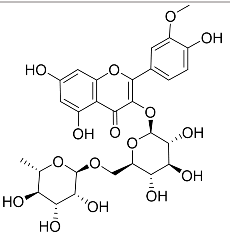 | Narcissoside |
| lightyellow | 63 | Caffeic acid linking central benzofuran or benzopyran unit | MOL007060 | 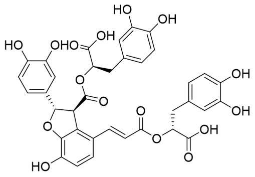 | Lithospermic acid B |
| midnightblue | 129 | Dammarane and oleanane type glycosides | MOL003034 | 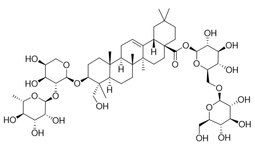 | Disacoside B |
| cyan | 38 | Caffeoylquinic acid | MOL001878 | 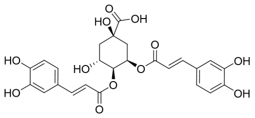 | Isochlorogenic acid C |
| red | 135 | Caffeic acid linking central sugar unit | MOL003333 | 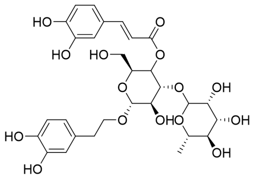 | Verbascoside |
| turquoise | 197 | Flavonoid aglycones | MOL000445 | 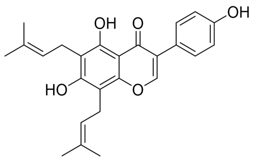 | 6,8-Diprenylgenistein |

| | | | | | |
|---|---|---|---|---|---|
| lightgreen | 33 | Phenyltetrahydrofuran derivatives | MOL000416 | 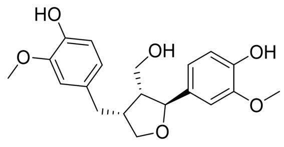 | Lariciresinol |
| grey60 | 37 | Gallic acid linking central sugar unit | MOL009133 | 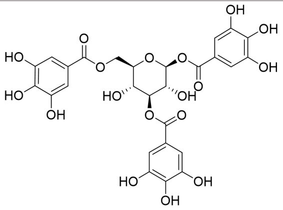 | 1,3,6-Trigalloylglucose |
| salmon | 128 | Hydrobenzofuran and phenylchromane derivatives | MOL000438 | 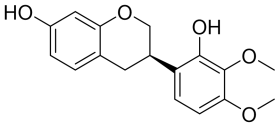 | Astraisoflavan |
| yellow | 166 | Dihydroanthracene derivatives | MOL006452 | 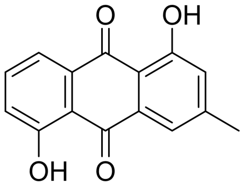 | 1,5-Dihydroxy-3-methylanthraquinone |
| tan | 40 | Cyclopenta naphthalene derivatives | MOL000290 | 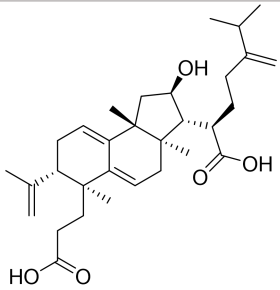 | Poricoic acid A |
| magenta | 264 | Dammarane and oleanane type aglycones | MOL001663 | 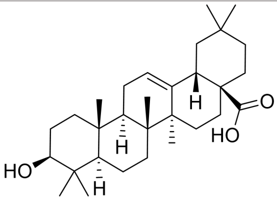 | Oleanic acid |
| greenyellow | 50 | Conjugated long-chain hydrocarbon analogs | MOL002742 | 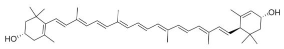 | Xanthophyll |

| | | | | | |
|---|---|---|---|---|---|
| blue | 229 | Glycosyladenosine and glycosylpyrimidine analogs | MOL001743 | 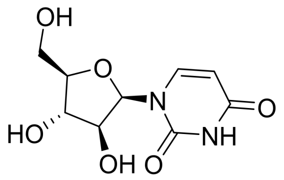 | 1-beta-D-Arabinofuranosyluracil |
| green | 273 | Long chain carboxylic acids and esters | MOL001281 | 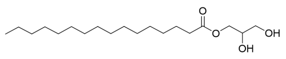 | 1-Monopalmitoyl-rac-glycerol |
| purple | 321 | Benzofuranone and benzopyranone derivatives | MOL002098 | 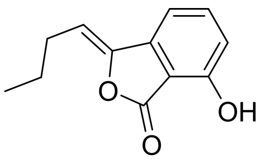 | 3-Butylidene-7-hydroxyphthalide |
| pink | 193 | Cinnamic acid derivatives | MOL010545 | 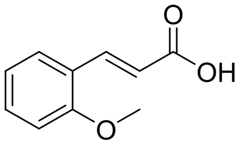 | (E)-2-methoxycinnamic acid |
| black | 693 | Cyclic and branched-chain hydrocarbons | MOL003978 | 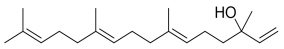 | Geranyl-linalool |
| lightcyan | 99 | Linear chain hydrocarbon | MOL010770 | 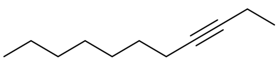 | 3-Undecyne |

**Supplementary Table 3. Molecular characteristics and representative compounds in 20 clusters.** The TMTP molecular library was clustered with a combination between Euclidean and Ward2 algorithms. 20 clusters were obtained, and the structural characteristics and representative compounds of each cluster were listed.

| Color | min | Q1 | median | Q3 | max | mean | sd | n | missing |
|---|---|---|---|---|---|---|---|---|---|
| royalblue | 0.645 | 0.732 | 0.806 | 0.837 | 0.944 | 0.793 | 0.076 | 19 | 0 |
| brown | 0.599 | 0.666 | 0.697 | 0.738 | 0.906 | 0.705 | 0.057 | 165 | 0 |
| lightyellow | 0.561 | 0.644 | 0.678 | 0.714 | 0.840 | 0.684 | 0.058 | 63 | 0 |
| midnightblue | 0.565 | 0.632 | 0.675 | 0.722 | 0.912 | 0.682 | 0.066 | 129 | 0 |
| cyan | 0.573 | 0.606 | 0.644 | 0.686 | 0.767 | 0.652 | 0.053 | 38 | 0 |
| red | 0.532 | 0.589 | 0.623 | 0.674 | 0.835 | 0.641 | 0.067 | 135 | 0 |
| turquoise | 0.399 | 0.588 | 0.604 | 0.628 | 0.782 | 0.609 | 0.037 | 197 | 0 |
| lightgreen | 0.557 | 0.582 | 0.596 | 0.648 | 0.735 | 0.614 | 0.047 | 33 | 0 |
| grey60 | 0.530 | 0.565 | 0.586 | 0.667 | 0.792 | 0.618 | 0.073 | 37 | 0 |
| salmon | 0.517 | 0.559 | 0.575 | 0.597 | 0.698 | 0.581 | 0.030 | 128 | 0 |
| yellow | 0.516 | 0.560 | 0.575 | 0.592 | 0.671 | 0.578 | 0.027 | 166 | 0 |
| tan | 0.534 | 0.552 | 0.569 | 0.605 | 0.629 | 0.577 | 0.030 | 40 | 0 |
| magenta | 0.489 | 0.549 | 0.561 | 0.578 | 0.662 | 0.564 | 0.027 | 264 | 0 |
| greenyellow | 0.474 | 0.508 | 0.546 | 0.569 | 0.643 | 0.545 | 0.044 | 50 | 0 |
| blue | 0.435 | 0.483 | 0.503 | 0.527 | 0.717 | 0.507 | 0.034 | 229 | 0 |
| green | 0.424 | 0.473 | 0.500 | 0.529 | 0.651 | 0.505 | 0.042 | 273 | 0 |
| purple | 0.425 | 0.473 | 0.492 | 0.519 | 0.613 | 0.498 | 0.035 | 321 | 0 |
| pink | 0.419 | 0.465 | 0.483 | 0.504 | 0.645 | 0.496 | 0.047 | 193 | 0 |
| black | 0.380 | 0.446 | 0.466 | 0.483 | 0.578 | 0.467 | 0.030 | 693 | 0 |
| lightcyan | 0.418 | 0.446 | 0.462 | 0.493 | 0.607 | 0.473 | 0.039 | 99 | 0 |

**Supplementary Table 4. Dominant clusters identification.** The dominant clusters were defined as which mean value of rank greater than 0.6.

| Molecule ID | clusters | $K_D$ (μM) |
|---|---|---|
| MOL003034 | midnightblue | 10.920 |
| MOL003331 | red | 1.212 |
| MOL003333 | red | 0.409 |
| MOL003686 | brown | 9.995 |
| MOL012143 | brown | 2.694 |
| MOL002761 | brown | 12.46 |
| MOL000415 | brown | 1.525 |
| MOL002693 | brown | 3.626 |
| MOL001543 | brown | 8.583 |
| MOL004958 | brown | 11.37 |
| MOL001878 | cyan | 0.047 |
| MOL004368 | brown | 9.977 |
| MOL000393 | midnightblue | 0.988 |
| MOL007260 | brown | 9.453 |
| MOL011620 | brown | 4.586 |
| MOL002067 | turquoise | 19.940 |
| MOL007113 | lightyellow | 15.040 |
| MOL007326 | cyan | 20.280 |
| MOL004515 | midnightblue | 7.199 |
| MOL007060 | lightyellow | 63.950 |
| MOL000422 | turquoise | 24.650 |

**Supplementary Table 5. SPR analysis of the interaction between TCM-derived components with 3CLpro of SARS-CoV-2.** Among the compounds form dominant clusters, 21 molecules showed high affinity for the SARS-CoV-2 3CLpro.

| Molecule ID | Clusters | $K_D$ (μM) | Predicted inhibitory activity |
|---|---|---|---|
| MOL003034 | midnightblue | 10.920 | 0.798 |
| MOL003331 | red | 1.212 | 0.540 |
| MOL003333 | red | 0.409 | 0.602 |
| MOL003686 | brown | 9.995 | 0.570 |
| MOL012143 | brown | 2.694 | 0.572 |
| MOL000415 | brown | 1.525 | 0.560 |
| MOL001543 | brown | 8.583 | 0.574 |
| MOL004958 | brown | 11.370 | 0.530 |
| MOL000393 | midnightblue | 0.988 | 0.782 |
| MOL011620 | brown | 4.586 | 0.534 |
| MOL004515 | midnightblue | 7.199 | 0.730 |

**Supplementary Table 6. 11 compounds that have shown the binding ability and predicted inhibitory activity with 3CLpro and their corresponding clusters.** After combined the molecules that were shown binding affinity with 3CLpro in the SPR analysis and the molecules that were predicted to be effective in DL, 11 compounds were considered to be promising candidate inhibitors. The value of predicted inhibitory activity is proportional to the intensity of the potential inhibitory activity, and it is considered as no inhibitory activity if it is less than 0.5.

| Residues<br>Compound | Narcissoside | Kaempferol-3-O-gentiobioside | Rutin | Vicenin-2 | Isoschaftoside |
|---|---|---|---|---|---|
| Thr25 | -0.827 | -0.825 | -0.925 | -1.771 | -0.643 |
| Thr26 | -0.248 | -0.298 | -0.920 | -1.996 | -1.439 |
| Leu27 | -1.258 | -1.447 | -1.363 | -2.238 | -2.762 |
| His41 | -2.279 | -2.613 | -2.795 | -1.792 | -1.983 |
| Cys44 | -0.079 | -0.866 | -0.589 | -0.076 | -0.023 |
| Thr45 | -0.727 | -0.779 | -0.623 | -0.066 | -0.012 |
| Ser46 | -0.900 | -1.066 | -0.203 | -0.008 | 0.026 |
| Met49 | -1.800 | -2.083 | -1.985 | -0.928 | -1.077 |
| Asn142 | -3.355 | -0.436 | -1.124 | -1.742 | -1.044 |
| Gly143 | -1.620 | -0.696 | -1.237 | -2.276 | -1.410 |
| Cys145 | -2.384 | -2.528 | -2.675 | -2.688 | -4.327 |
| His163 | -0.476 | -0.605 | -1.488 | -0.363 | -1.223 |
| His164 | -1.209 | -0.621 | -0.292 | -0.209 | -0.472 |
| Met165 | -2.636 | -2.287 | -1.089 | -1.220 | -2.473 |
| Asp187 | -0.578 | -1.188 | -0.370 | -0.355 | -0.316 |
| Gln189 | -1.567 | -1.347 | -1.209 | -0.308 | -0.740 |

**Supplementary Table 7. Residues contribution to the total binding energy during the MD simulation.** Residues with a high contribution to the total binding energy were listed during the MD simulation of the 5 NPs-3CLpro complexes. The energy values of the residues are in kcal/mol.

| Residues Compound | Narcissoside | Kaempferol-3-O-gentiobioside | Rutin | Vicenin-2 | Isoschaftoside |
|---|---|---|---|---|---|
| His41 | -2.279 | -2.613 | -2.795 | -1.792 | -1.983 |
| Val42 | -0.191 | -0.440 | -0.314 | -0.287 | -0.178 |
| Ile43 | -0.029 | -0.107 | -0.088 | 0.022 | 0.045 |
| Cys44 | -0.079 | -0.866 | -0.589 | -0.076 | -0.023 |
| Thr45 | -0.727 | -0.779 | -0.623 | -0.066 | -0.012 |
| Ser46 | -0.900 | -1.066 | -0.203 | -0.008 | 0.026 |
| Glu47 | 0.262 | 0.230 | 0.011 | 0.060 | 0.063 |
| Asp48 | -0.067 | -0.111 | -0.306 | -0.015 | -0.008 |
| Met49 | -1.800 | -2.083 | -1.985 | -0.928 | -1.077 |
| Total energy | -5.810 | -7.837 | -6.892 | -3.092 | -3.147 |

**Supplementary Table 8. The total energy of residues based on the region of His41 to Met49.** The total energy contribution of His41 to Met49 were demonstrated in 5 inhibitors-3CLpro complexes. The energy values of the residues are in kcal/mol.
